# Separation and Determination of Fatty Acids from Lipid Fractions by High-Performance Liquid Chromatography: Cholesterol Esters of Umbilical Cord Arteries

**DOI:** 10.1080/15376510701623912

**Published:** 2008-06-23

**Authors:** Lech Romanowicz, Stefan Jaworski, Zofia Galewska, Tomasz Gogiel

**Affiliations:** Department of Medical Biochemistry and Department of Gynecology, Medical University of Białystok, Białystok, Poland

**Keywords:** Cholesteryl Esters, Fatty Acids, HPLC, Preeclampsia, Umbilical Cord Artery

## Abstract

Preeclampsia is accompanied by an extensive remodeling of the extracellular matrix of umbilical cord. It is associated with an increase in collagen content in the umbilical cord artery. Furthermore, preeclampsia distinctly reduces proteolytic and gelatinolytic activity, especially after activation with various agents.

We decided to develop a method for separation and determination of fatty acids from different tissues by high-performance liquid chromatography. That method allowed us to determine cholesteryl ester composition and content in umbilical cord arteries. Studies were performed on the umbilical cord arteries taken from 10 newborns delivered by healthy mothers and 10 newborns delivered by mothers with preeclampsia. Cholesteryl esters were isolated by thin layer chromatography. Fatty acids were liberated by basic hydrolysis and analyzed by HPLC of their p-bromophenacyl derivatives using detection at 254 nm. It was found that saturated fatty acids were the main group of fatty acids incorporated to cholesteryl esters in all control and preeclamptic umbilical cord arteries. Preeclampsia caused a significant increase in cholesteryl ester content in the umbilical cord arteries. An increase of neutral lipid content in vessel walls of newborns delivered by mothers with preeclampsia may be one of the factors that evoke the initiation of hypertension in utero and its amplification throughout childhood and adult life. The described method reduces time and cost consumption and allows us to determine almost all fatty acids forming cholesteryl esters contained in the tissue sample.

## INTRODUCTION

Lipids are a huge and molecularly diversified group of compounds that is present in all living organisms. They serve several important roles in cells, such as components forming biological membranes or participating in signal transduction into the cell. Fatty acid composition is one of the most important attributes of lipid fractions isolated from different tissues. After isolation of whole lipids from biological material under investigation, studied lipid fraction is separated from others by using the method of thin layer chromatography (Sonda et al. 2001) or solid phase extraction (Pernet et al. 2006). In order to determine their exact characterization, fatty acids contained in investigated lipid fraction should be liberated by basic hydrolysis (Engelmann et al. 1988) or methylation (Nawrocki and Górski 2004). Qualitative and quantitative analysis of those fatty acids could be made by gas chromatography (Nawrocki and Górski 2004) or high-performance liquid chromatography (Engelmann et al. 1988).

It is commonly known that the vascular system of mother and placenta plays an important role in the intrauterine development of the fetus. Preeclampsia is the most common pregnancy-associated pathological syndrome (Sankaralingam et al. 2006). It was found in our previous studies that preeclampsia is accompanied by an extensive remodeling of extracellular matrix of the umbilical cord (Bańkowski 1999; Pawlicka et al. 1999; Romanowicz et al. 1994). There are only few data on phospholipids of umbilical cord vessels (Velzing-Aarts et al. 1999) and there is not much information about neutral lipids. Therefore, we decided to isolate, fractionate, and determine cholesteryl esters of umbilical cord arteries taken from newborns of healthy mothers and those with preeclampsia. For implementation of that it was decided to develop separation and determination of fatty acids with the use of HPLC.

## MATERIALS AND METHODS

### Tissue Material

Studies were performed on the umbilical cord arteries (UCAs) taken from 20 newborns. In all cases 20-cm-long sections of UCAs were excised beginning from their placental end and carefully separated from the surrounding Wharton's jelly.

The control material was taken from 10 newborns delivered by healthy mothers aged 23 to 38 with normal blood pressure (systolic 100–139 mm Hg, diastolic 70–89 mm Hg). The mothers presented no symptoms of edema or renal failure. The mean body weight of the newborns was 3,505 ± 340 g.

The 10 investigated newborns were delivered by mothers aged 22 to 34 with preeclampsia, diagnosed according to the criteria accepted by the Organisation Gestosis (Rippmann 1971). All patients demonstrated an elevation of blood pressure (systolic > 140 mm Hg, diastolic >90 mm Hg) and proteinuria (greater than trace). All cases of patients with cardiovascular, renal, and metabolic diseases were excluded. The mean body weight of these newborns was equal to 3,185 ± 514 g.

### Lipid Isolation

Lipids were isolated according to the method described by van der Vusse and colleages with some minor modifications (Folch et al. 1957; van der Vusse et al. 1983). One hundred milligrams of UCA wall was immersed in 0.9% NaCl for rinsing from blood. Then tissue was cut into small pieces and immersed in 2 mL of cold methanol (−20° C) containing 600 mg of butylated hydroxytoluene as an antioxidant. Next, tissue was homogenized three times with knife homogenizer for 45 sec followed by three times sonification for 15 sec in ice-water bath. Four milliliters of chloroform containing 1 μg of nonadecanoic acid and 20 μg of triacylglycerol with three residues of the same acid as inner standards was added. The homogenate was left at 2 to 8°C until the next day. After adding 1.5 mL of deionized water, sample was shaken for 1 min and then centrifuged at 3,000 rpm for 10 min. Lower chloroform phase was transferred into a new tube and solvent was evaporated with nitrogen stream at 37°C. Dry sediment was dissolved in 0.1 mL of chloroform:methanol mixture (2:1, v/v).

### Thin Layer Chromatography

Cholesteryl esters were separated by thin layer chromatography (Nawrocki and Górski 2004; Sonda et al. 2001). After putting the whole sample on plate with silica gel, it was dried on air for about 15 min. Then ascending chromatography was made in mixture containing heptane:isopropyl ether:acetic acid (60:40:3, v/v/v). Separation was stopped when the front of the solvents was a half-centimeter below the upper edge of the plate. Gel was dried completely at rt, sprinkled with 0.2% methanol solution of 2',7'-dichlorofluorescein, and fixed in ammonia vapor in order to visualize lipid fractions in UV light (254 nm). In conditions described above cholesteryl esters were the fastest gone lipid fraction. The exact spot of cholesteryl esters was marked. Gel with that spot was scrubbed from the plate. Cholesteryl esters were eluted with 2 mL of diethyl ether at 2 to 8°C for 1 h. The sample was then shaken for 1 min. Solution over the sediment was transferred to a new tube. The elution procedure was repeated once more without waiting for 1 h. Both solutions were combined and dried with nitrogen stream at 37°C.

### Alkaline Hydrolysis

Immediately, dry sample was dissolved in 1 mL of 2 M KOH solution in 80% methanol and hydrolyzed according to Engelmann et al. (1988). Hydrolysis was carried out at 80°C for 1 h with constant shaking in closed tubes. Then the solution was cooled to rt and neutralized with 1 M methanol solution of HCl in the presence of phenolphthalein. Solvent was evaporated with nitrogen stream at 37°C. Potassium salts of fatty acids were extracted twice with the use of 1 mL of chloroform by shaking for 1 min. Chloroform fractions were combined and evaporated with nitrogen stream at 37°C. Solid sample was dissolved in 100 μL of chloroform following by adding 100 μL of acetonitrile. Sample prepared in such a way was ready for derivatization needed for fatty acid separation by high-performance liquid chromatography. Samples could be stored at 2 to 8°C overnight.

### Derivatization

Para-bromophenacyl esters of free fatty acids after hydrolysis were prepared as described by Tsikas and coworkers (2003). The sample was mixed with 48 μL of 0.01 M of KOH water solution, 56 μL of 2,3,11,12-dicyclohexano-1,4,7,10,13,16-hexaoxacyclooctadecane solution in acetonitrile (1 mg/mL), and 80 μL of 4-bromophenacyl bromide solution in acetonitrile (10 mg/mL). Sample was incubated at 80°C in water bath with constant shaking for 15 min in well-closed tubes. Next, sample was cooled to rt followed by vacuum-dry solvents at 37°C. Dry sediment was dissolved in 292 μL of acetonitrile. Samples could be stored at 2 to 8°C overnight.

### Determination

Dissolved sample was directed to separation and determination of particular fatty acid by reversed-phase high-performance liquid chromatography (LaChrom System, Merck Hitachi) (Engelmann et al. 1988). LiChroCART RP-18 column (5 μm, 25 cm x 4 mm) with LiChroCART RP-18 guard column (Merck, Darmstadt, Germany) was used. Separation was carried out at 40°C with the constant flow rate 1.6 mL/min. The gradient solvent mixture execution is shown in Table 1.

**TABLE 1:** The gradient solvent mixture execution.

Time (min)	Methanol (%)	0.5 mM TEAP buffer pH 5.6 (%)	H_2_O (%)	Acetonitrile (%)
0	70	11	0	19
22	70	11	0	19
24	70	4	0	26
30	69	0	5	26
40	0	0	5	95
45	70	11	0	19

0.5 mM TEAP buffer pH 5.6: 0.5 mM phosphoric acid, pH brought to point with triethylamine.

Absorbance was measured at the wavelength of 254 nm with the use of DAD L-7455 detector (Merck Hitachi). Data were handled with D-7000 HSM Chromatograph Data Station Software, version 4.0 (Merck Hitachi). The method described above allowed us to separate and to determine fatty acids as specified in Table 2.

**TABLE 2:** Separated and determined fatty acids by the method described above

Carbon atom number and double bond number	Fatty acid
Saturated fatty acids (SAFAs)	
C 12:0	lauric
C 14:0	myristic
C 16:0	palmitic
C 18:0	stearic
C 20:0	arachidic
C 22:0	behenic
Monounsaturated fatty acids (MUFAs)	
C 14:1	myristoleic
C 16:1	palmitoleic
C 18:1	oleic
C 24:1	nervonic
Polyunsaturated fatty acids (PUFAs)	
C 18:2	linoleic
C 18:3	linolenic
C 20:4	arachidonic
C 20:5	eicosapentaenoic
C 22:6	docosahexaenoic

### Statistical Analysis

Mean values from 10 assays ± standard deviations (SDs) were calculated. Particular fatty acid content in cholesteryl esters from umbilical cord artery wall was expressed in mol%. The results were submitted to statistical analysis with the use of Student's *t*-test, accepting *p* < 0.05 as significant.

## RESULTS

Thin layer chromatography of isolated lipids from umbilical cord arteries allowed separating cholesteryl esters from other neutral lipids. After elution from gel, basic hydrolysis of those esters for liberation of potassium salts of fatty acids, and their derivatization, they were separated and determined by HPLC. The used method allowed us to isolate saturated fatty acids (SAFAs) lauric, myristic, palmitic, stearic, arachidic, and behenic acid; monounsaturated fatty acids (MUFAs) myristoleic, palmitoleic, oleic, and nervonic acid; and polyunsaturated fatty acids (PUFAs) linoleic, linolenic, arachidonic, eicosapentaenoic, and docosahexaenoic acid.

As can be seen from Table 3A, stearic acid and palmitic acid predominate among SAFAs, oleic acid in MUFAs, and linoleic acid among PUFAs in control UCA wall. Preeclampsia evokes more than a 50% decrease in their content. Lauric acid is present in the highest quantity of all SAFAs in preeclamptic UCA wall. The highest amount of all investigated fatty acids is observed for docosahexaenoic acid in preeclamptic material.

**TABLE 3A:** Fatty acid content in cholesteryl esters of umbilical cord arteries

Fatty acid	Control UCA wall [mol%]	Preeclamptic UCA wall
SAFA		
C 12:0	5.05 ± 0.82	15.52 ± 0.99
C 14:0	3.09 ± 0.41	0.89 ± 0.29
C 16:0	13.14 ± 1.03	5.73 ± 0.94
C 18:0	24.90 ± 2.05	12.00 ± 1.98
C 20:0	1.13 ± 0.17	6.71 ± 0.21
C 22:0	n.d.	1.62 ± 0.19
MUFA		
C 14:1	5.47 ± 0.22	1.45 ± 0.17
C 16:1	2.62 ± 0.18	n.d.
C 18:1	23.91 ± 1.96	9.75 ± 1.78
C 24:1	n.d.	1.62 ± 0.17
PUFA		
C 18:2	6.02 ± 0.59	4.11 ± 0.51
C 18:3	1.05 ± 0.20	7.35 ± 0.29
C 20:4	4.91 ± 0.31	1.77 ± 0.25
C 20:5	5.03 ± 0.77	13.21 ± 0.82
C 22:6	3.70 ± 0.52	18.28 ± 0.61

n.d., not detected.

The percentage of SAFA, MUFA, and PUFA content of cholesteryl esters in umbilical cord arteries is present in Table 3B. In control subjects SAFAs are present in the highest amount in comparison to MUFAs and PUFAs. Preeclampsia is associated with the significant decrease in SAFA proportional content in UCA wall. Also, relative MUFA content is three times lower in preeclamptic UCA in comparison to control material. On the other hand, PUFA content is more than two times higher in preeclamptic UCA wall (Table 3B).

**TABLE 3B:** Percentage of saturated (SAFA), monounsaturated (MUFA), and polyunsaturated (PUFA) fatty acids from cholesteryl esters of umbilical cord arteries

	Control UCA wall	Preeclamptic UCA wall
SAFA	47.31	42.47
MUFA	32.00	12.82
PUFA	20.71	44.72

Total content of cholesteryl esters in control UCA is 4.22 ± 0.76 μmol/g of tissue, whereas in preeclamptic UCA wall, it reaches more then two times higher value: 9.35 ± 1.02 μmol/g of tissue.

## DISCUSSION

The umbilical cord forms the connection between the placenta and the fetus. The cross section of the umbilical cord shows a specific gross morphology of one vein and two arteries surrounded by a distinct connective tissue region called Wharton's jelly. Both arteries lead venous blood from the fetus to the placenta. The umbilical cord vein pipes off fetal blood from the placenta to the fetus. Major exchange of all substances between fetus and mother occurs by fetal blood in placenta. Therefore, umbilical cord arteries and vein walls have continuous contact with all substances cruising in fetal blood.

Genetic, immunologic, and dietary factors may be involved in the pathogenesis of preeclampsia. It is accompanied by significant morphological and functional alterations in the arterial walls of the uterus and placenta (Bańkowski et al. 1993). Our previous studies have shown that preeclampsia is accompanied by an extensive remodeling of extracellular matrix of the umbilical cord. The umbilical cord arteries of newborns delivered by mothers with preeclampsia contain more than twice the amount of collagen and markedly less elastin in comparison to corresponding arteries of newborns delivered by healthy mothers (Bańkowski 1999; Pawlicka et al. 1999). The changes in collagen composition are accompanied by an early reduction of hyaluronic acid in the umbilical cord arterial wall and its replacement by sulphated glycosaminoglycans (Romanowicz et al. 1994).

Cholesteryl esters belong to the group of neutral lipids. They are localized intracellularly only. They play a role as a reservoir of cholesterol and different fatty acids, which can be liberated successively, depending on cell wants. It is of interest that preeclampsia is accompanied by an accumulation of cholesteryl esters in UCA wall in comparison to UCA wall of newborns delivered by healthy mothers. Such an accumulation of neutral lipids in artery wall may evoke disadvantageous changes in cell metabolism, which could be responsible for the remodeling of the umbilical cord vessels, especially extracellular matrix. Such changes may be a reason of a decrease in blood flow in the fetus of woman with preeclampsia.

It is commonly known that preeclampsia is a syndrome that results in a reduction in birth weight of newborns. In fetuses with retarded growth Doppler ultrasound has shown evidence of increased peripheral resistance to blood flow in the descending aorta (Griffin et al. 1983). According to Gonzalez et al. (2007), some cases of intrauterine growth retardation are associated with maternal hypertension. It is apparent from the reports of several authors that low birth weight for the period of gestation may be a predictor of raised blood pressure in childhood and adult life (Eriksson et al. 2000; Fattal-Valevski et al. 2001; Law et al. 2002). It may be supposed that preeclampsia is a factor that evokes the initiation of hypertension in utero and its amplification throughout childhood and adult life.

Our results show that the method for isolating lipid fraction and liberating and determining fatty acids from that fraction, described in this paper, can be used for different tissue samples. Lipids are isolated from fresh tissue by homogenization and extraction in methanol:chloroform mixture. Particular lipid fraction was obtained by thin layer chromatography on silica gel. Fatty acids from lipid fraction were liberated by basic hydrolysis and converted into p-bromophenacyl derivatives. Those derivatives were separated and determined by reverse-phase high-performance liquid chromatography in gradient mode of solvents. The results obtained are comparable to those of gas chromatography (Bakewell et al. 2006; Decsi et al. 2001). Both separating fatty acid methods need earlier preparation of investigated samples. The direct method for separation and determination of fatty acids from particular lipid fraction is lacking. The presented HPLC method consumes less time (e.g., one separation process lasts 45 min instead of 2 h as occurs in gas chromatography). Application of TEAP buffer (containing phosphoric acid and triethylamine) as one of the solvents in gradient mode for HPLC separation of fatty acid derivatives improves separating method because it does not precipitate and stop the flow through the column like inorganic buffer containing potassium phosphate.

The described method of fatty acid determination allows shortening time consumption for separation of particular sample and reagent saving, so it reduces cost of each sample determination.
